# Ultrasonic Assisted-Reflux Synergistic Extraction of Camptothecin and Betulinic Acid from *Camptotheca acuminata* Decne. Fruits

**DOI:** 10.3390/molecules22071076

**Published:** 2017-06-27

**Authors:** Chunying Li, Yukun Zhang, Chunjian Zhao, Yujiao Ni, Kaiting Wang, Jingjing Zhang, Wenyan Zhao

**Affiliations:** 1 Key Laboratory of Forest Plant Ecology, Ministry of Education, Northeast Forestry University, Harbin 150040, China; nefujane@aliyun.com (C.L.); klp14zyk@nefu.edu.cn (Y.Z.); klp14nyj@nefu.edu.cn (Y.N.); klp14wkt@nefu.edu.cn (K.W.); klp15zjj@nefu.edu.cn (J.Z.); klp15zhwy@nefu.edu.cn (W.Z.); 2 Collaborative Innovation Center for Development and Utilization of Forest Resources, Harbin 150040, China

**Keywords:** *Camptotheca acuminata* Decne., CPT, BA, UARSE

## Abstract

A novel and efficient ultrasonic assisted-reflux synergistic extraction (UARSE) method for extracting camptothecin (CPT) and betulinic acid (BA) from *Camptotheca acuminata* Decne. fruits has been developed in this study. The advantages of the ultrasonic and reflux extraction methods have been combined in the UARSE method and used to extract CPT and BA for the first time. The parameters influencing the efficiency of UARSE were optimized using the Box-Behnken design (BBD) to obtain the maximum extraction yield of CPT and BA. The optimal extraction conditions were as follows: 225 W for the ultrasonic power; 24 min for the extraction time; and 32 mL/g for the liquid–solid ratio. The extraction yields obtained by UARSE were 2.386 ± 0.112 mg/g for CPT and 17.192 ± 0.808 mg/g for BA, which were 1.43-fold and 1.33-fold, respectively, higher than by using heating reflux extraction (HRE) and ultrasonic-assisted extraction (UAE). In addition, the 24-min extraction time using UARSE was 80% and 60% less than those provided by HRE and UAE, respectively. Therefore, UARSE can be considered a rapid and efficient method for extracting CPT and BA from the fruits of *C. acuminata* Decne.

## 1. Introduction

**Figure 1:**
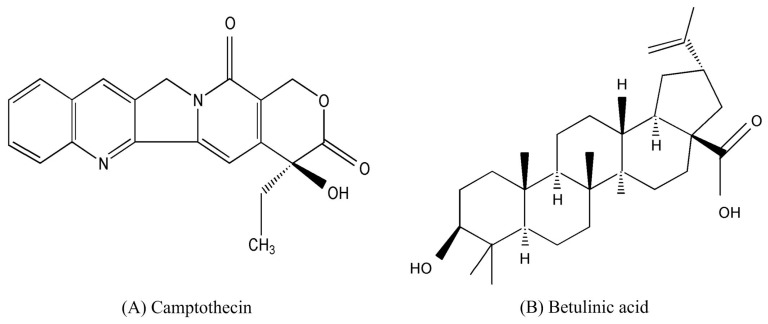
Chemical structures of (**A**): camptothecin (CPT), and (**B**): betulinic acid (BA).

*Camptotheca acuminata* Decne. (Nyssaceae) is an indigenous Chinese plant species widely grown in Asia. Because different parts of this plant are rich in natural active compounds, such as alkaloids, glycosides, and flavonoids, it has attracted much scientific attention [1,2]. Camptothecin (CPT, Figure 1), the main anti-cancer monoterpene indole alkaloid, occurs naturally in *Camptotheca acuminata* Decne. In the 1980s, its anti-tumor activity, based on its ability to inhibit topoisomerase I, an enzyme involved in DNA replication, was discovered [3,4]. CPT has been used clinically for treating ovarian and small lung cancers [5] and has also exhibited potential anti-viral (HIV and herpes), anti-psoriatic, and anti-fungal activities [6]. Betulinic acid (3β-hydroxy-lup-20(29)-en-28-oic acid, BA, Figure 1), a natural pentacyclic triterpene also widely distributed in *Camptotheca acuminata* Decne., has antitumor, anti-HIV, anti-inflammatory, and antibacterial activities [7,8,9,10].

Details of different extraction methods for obtaining CPT or BA, such as stirring extraction, Soxhlet extraction, and heating reflux extraction (HRE), have been reported [11,12,13,14,15,16,17,18,19]. However, the main disadvantages of these conventional extraction techniques are the long extraction times and low yields [19,20,21]. Recent studies have shown the great potential of ultrasonic-assisted extraction (UAE) for efficiently obtaining specific active natural compounds from biomaterials [22,23,24].

The present study aims to develop an ultrasonic assisted-reflux synergistic extraction (UARSE) method for extracting CPT and BA from *Camptotheca acuminata* Decne. fruits. This novel extraction method combines the advantages of both ultrasonic-assisted extraction (UAE) and heating reflux extraction (HRE) to dramatically reduce the extraction time and increase the extraction yield of the target compounds. The study will investigate how to enhance the extraction process of this innovative method by studying various parameters, such as ultrasonic power, extraction time, and liquid–solid ratio to obtain the optimum processing conditions. The advantages of UARSE will be compared with those of the UAE and HRE methods and the optimized conditions for UARSE established using a Box-Behnken design (BBD) combined with response surface methodology (RSM). The ultrastructure of the plant materials obtained by different extraction methods will also be observed using scanning electron microscopy (SEM).

## 2. Results and Discussion

### 2.1. Effect of Independent Variables on Extraction Yield

#### 2.1.1. Effect of Liquid–Solid Ratio

**Figure 2:**
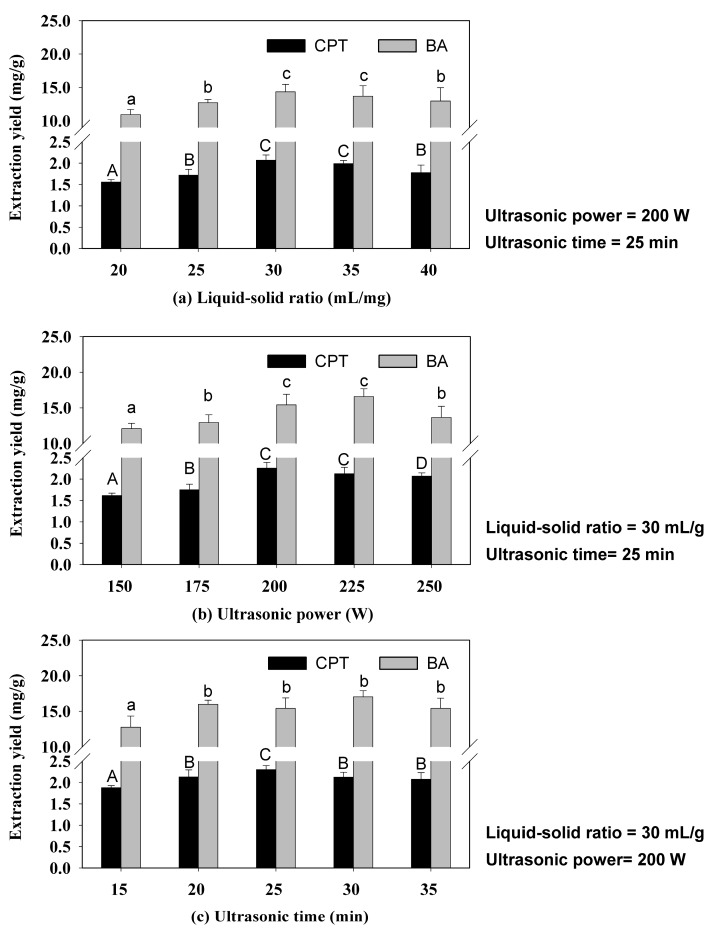
Effect of liquid–solid ratio (**a**); ultrasonic power (**b**) and ultrasonic time (**c**) on extraction yield of target compounds. Values are mean ± standard error (*n* = 3 replicates). Columns with the same letters are not significantly different (*p* < 0.05).

The effect of varying the liquid–solid ratios on extraction yield of CPT and BA from 20 to 40 mL/g was investigated for optimizing the processing conditions (Figure 2a). The extraction yield of the two target compounds increased as the liquid–solid ratio increased from 20 to 30 mL/g, reaching a maximum at 30 mL/g. At ratios above 30 mL/g, the yields of the target compounds no longer increased. Hence, a liquid–solid ratio of 30 mL/g was selected for the further optimization studies.

#### 2.1.2. Effect of Ultrasonic Power

The level of ultrasonic power controls the intensity of cavitation, which helps to release the target compounds from the plant matrix. The effects of varying the ultrasonic power from 150 to 250 W on the extraction yields of CPT and BA at condition of the same time and liquid–solid ratio were tested. Figure 2b shows that the yields of CPT and BA increased gradually when the ultrasonic power increased from 150 to 200 W. The powerful ultrasound probably caused a large number of cavitation bubbles to form, which increased the mass transfer and interactions between the solvent and the plant matrix [25]. The collapse of cavitation bubbles near tissue surfaces can rupture the cell walls which could increase the penetration of solvent into the tissue matrix, leading to a gradual increase in extraction yield [26]. In addition, Figure 2b shows that the yields of the two target compounds were not significantly different between power settings of 200 and 225 W. With the increase of ultrasonic power from 225 W to 250 W, the extraction yields of BA and CPT decreased slightly. This reduction may have been the result of the target compounds degrading under the high ultrasonic power [27]. The extraction process under 225 W of ultrasonic power consumes less energy than 225 W; therefore, 200 W was selected as the optimal ultrasonic power for extracting BA and CPT.

#### 2.1.3. Effect of Ultrasonic Time

The ultrasonic extraction time is an important function during solvent extraction [28]. The influence of time on the yield of the two compounds was assessed over a range of 15–35 min using 200 W of ultrasonic power under the same liquid–solid ratio conditions. Figure 2c shows that the extraction yields of CPT and BA clearly increased for times up to 20 min, then did not change significantly. This phenomenon may be explained by the ultrasonic waves induced at the beginning of ultrasonic processing causing chaotic vibrations at the solvent–solid interface. These vibrations could then disrupt the cells and speed up the release and diffusion of the target compounds, thus improving the extraction yields of the target compounds markedly to reach a maximum value. For longer ultrasonic time, the target components would no longer be released, so extraction yields would not have changed significantly. Therefore, 20 min was selected as the ultrasonic time for the further experiments.

### 2.2. Optimization of Extraction Conditions of UARSE

Based on these single factor experiments, a Box–Behnken design (BBD) combined with RSM was used to investigate the interaction of the experiment conditions and to optimize the extraction conditions for the target compounds. The experimental conditions and the results of 17 runs using the BBD design are shown in Table 1 with results performed in triplicate. The yield of CPT (Y_1_) and BA (Y_2_) was a function of three independent variables (liquid–solid ratio, X_1_; ultrasonic time, X_2_; and ultrasonic power, X_3_). By applying multiple regression analysis to the experimental data, the response variable and the test variables were found to be related by the following second-order polynomial expressions:

Y_1_ = 2.31 + 0.092X_1_ + 0.083X_2_ + 0.24X_3_ + 6.5 × 10^−3^X_1_X_2_ + 0.027X_1_X_3_ + 0.13X_2_X_3_ − 0.52X_1_^2^ − 0.3X_2_^2^ − 0.16X_3_^2^ (1)

Y_2_ = 16.46 +1.13X_1_ + 0.85X_2_ + 1.67X_3_ − 0.062X_1_X_2_ + 0.37X_1_X_3_ + 0.064X_2_X_3_ − 1.91X_1_^2^ − 0.75X_2_^2^ − 1.52X_3_^2^ (2)

The statistical significance and adequacy of the regression model were evaluated by the *F*-test and *p*-value. The larger the absolute *F*-value and the smaller the *p*-value, the more significant was the corresponding model term. The analysis of variance (ANOVA) for the response surface quadratic polynomial model is summarized in Table 2. From the statistical analysis, desirable determination coefficients (*R*^2^), 0.9858 for CPT and 0.9912 for BA, were obtained for the calculated model; the lack of fit was not significant (*p* > 0.05); and the highly significant level obtained for the model (*p* < 0.0001) indicated that it was precise and applicable. The combination of the *p*-value of the model, the lack of fit and determination coefficients indicated that the model equations were adequate for reasonably predicting the yield of the two target compounds. Table 2 shows that the linear coefficient (X_3_), and quadratic terms (X_1_^2^, X_2_^2^) had a significant effect on the extraction yields of CPT and BA (*p* < 0.0001).

**Figure 3:**
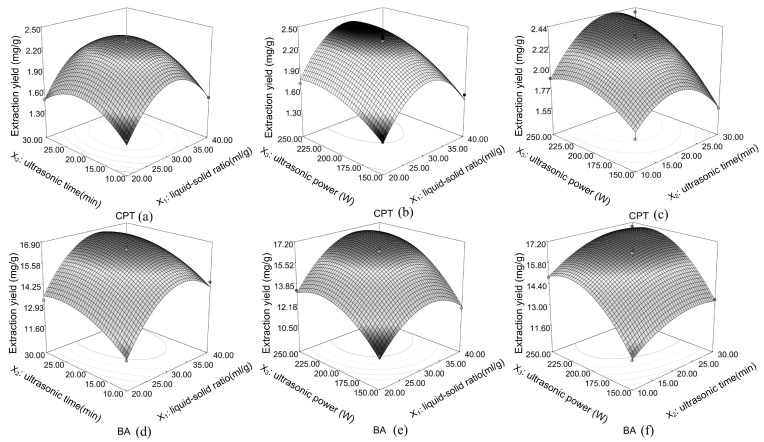
Response surface plots showing the effects of variables (X_1_: liquid–solid ratio, mL/g; X_2_: ultrasonic time, min; and X_3_: ultrasonic power, W) on the extraction yields of CPT (**a**–**c**) and BA (**d**–**f**).

The 3D response surface visualizes the relationship between responses and experimental levels of each variable with the contour profiles indicating the significance of the interactions between variables. The effects of the liquid–solid ratio, ultrasonic time and ultrasonic power on the extraction yield of the two target compounds, as well as their interactions, are shown in Figure 3.

Based on Equations (1) and (2), the optimum extraction conditions (independent variables) proposed by the Design Expert software were identified: the maximal CPT yield was obtained at a liquid–solid ratio of 31.15 mL/g, an ultrasonic time of 23.42 min, using an ultrasonic power of 246.37 W. Similarly, the maximal BA yield was obtained at a liquid–solid ratio of 33.47 mL/g, an ultrasonic time of 25.77 min, using an ultrasonic power of 230.06 W. Considering the yield and actual operation, the liquid–solid ratio, ultrasonic time, and ultrasonic power were modified to 32 mL/g, 24 min and 225 W, respectively. Under these conditions, the experimental values of CPT and BA yields (2.386 ± 0.112 mg/g and 17.192 ± 0.808 mg/g) obtained by UARSE agreed with the predicted values with only a low deviation (1.15%), thus indicating that the predictive performance of the established RSM models was reliable.

### 2.3. Comparison of Different Extraction Methods

**Figure 4:**
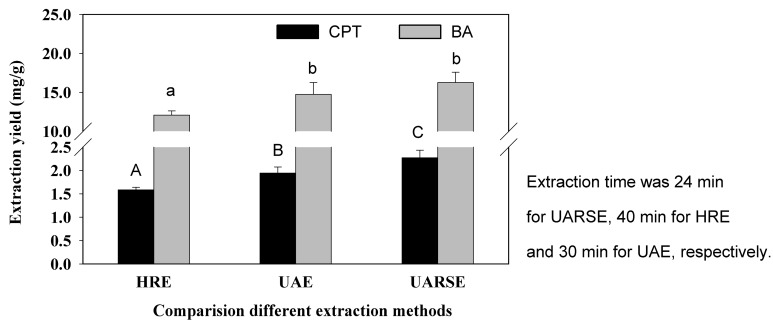
Comparison of different methods for extracting CPT and BA from *C. acuminata* Decne. fruits. Columns with the same letter are not significantly different (*p* < 0.05).

The UARSE, HRE and UAE methods were compared (Figure 4). This indicated that UARSE provided the highest extract yields of CPT and BA (2.386 ± 0.112 mg/g and 17.192 ± 0.808 mg/g, respectively). The yields of CPT and BA provided by UAE (2.036 ± 0.094 mg/g CPT and 15.804 ± 0.727 mg/g, respectively) and HRE (1.624 ± 0.070 mg/g and 12.457 ± 0.536 mg/g, respectively) were lower. In addition, the extraction time using UARSE required for the equilibrium yields of CPT and BA was only 24 min, which was 80% and 60% less than those for HRE and UAE, respectively.

### 2.4. Scanning Electron Microscopy (SEM)

**Figure 5:**
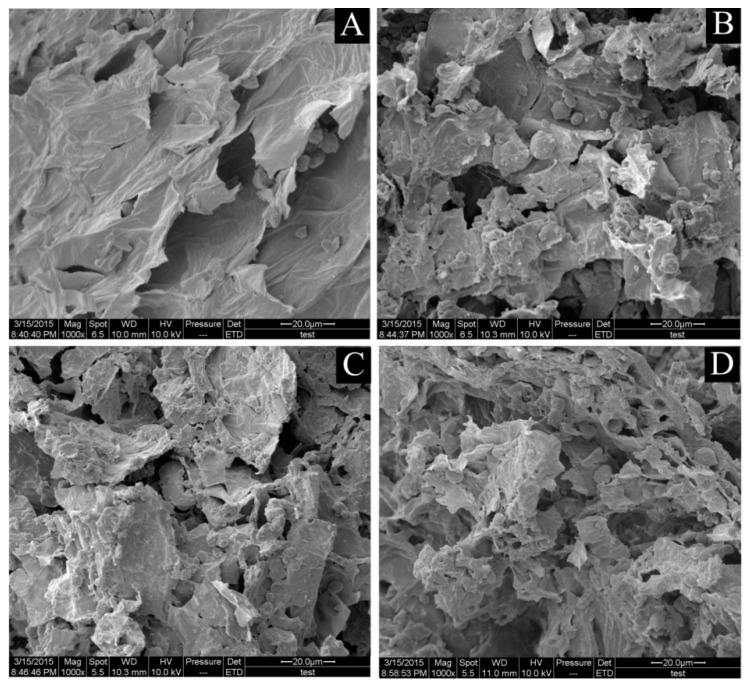
Scanning electron microscopic images of *C. acuminata* Decne. fruit samples. (**A**): Raw materials; (**B**–**D**) Show samples treated by HRE, UAE, and UARSE, respectively.

To investigate the correlation between extraction yield and cell wall breakage, scanning electron microscopy (SEM) was used to observe the structure of untreated samples and those extracted using the different extraction methods (UARSE, HRE and UAE). These different methods produced great physical changes on the tissue of the *Camptotheca acuminata* Decne. fruits (Figure 5A–D, respectively). Figure 5A clearly shows that the external surface of the untreated sample tissues was intact and smooth. After HRE treatment, some cells were slightly damaged (Figure 5B) with comparatively more being destroyed by UAE (Figure 5C), but most of the cells were completely disrupted and collapsed after UARSE treatment (Figure 5D). This indicated that UARSE ruptured cell walls more effectively, thus resulting in a higher extraction yield.

## 3. Materials and Methods

### 3.1. Plant Materials and Chemicals

The *Camptotheca acuminata* Decne. fruits were collected from Jintang County in Sichuan Province (China). The materials were dried in the shade, broken down to a powder using a disintegrator (HX-200A, Yongkang Hardware and Medical Instrument Plant, Yongkang, China), passed through a stainless-steel sieve (60–80 mesh) then stored in closed desiccators at 4 °C until use CPT (98%) and BA standards (98%) were purchased from Nanjing Spring & Autumn Biological Engineering Co. Ltd. (Nanjing, China). HPLC grade methanol was purchased from J&K Chemical Ltd. (Beijing, China). Deionized water for HPLC was purified using a Milli-Q Water Purification system (Millipore, Billerica, MA, USA). Other analytical reagents were purchased from the Tianjin Kermel Chemical Reagent Co. Ltd. (Tianjin, China). All solutions and samples prepared for analysis were filtered through a 0.45-μm nylon membrane (Guangfu Chemical Reagents Co., Tianjin, China).

### 3.2. Apparatus

**Figure 6:**
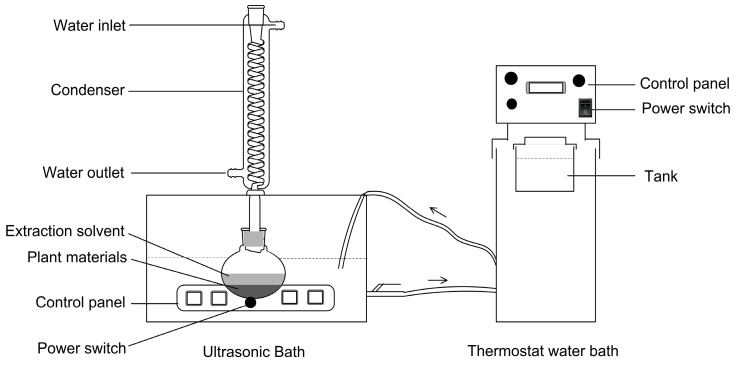
Schematic representation of the UARSE device.

**Figure 7:**
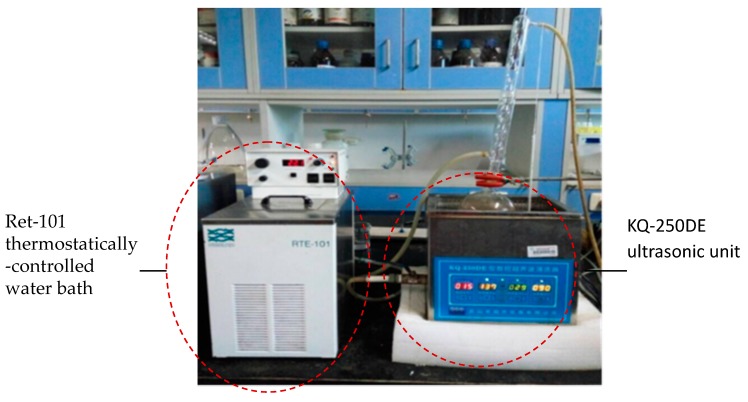
A picture of the UARSE device.

The ultrasonic assisted-reflux synergistic extraction (UARSE) device was made up of an ultrasonic unit and a thermostatic water bath (Figure 6 and Figure 7). The KQ-250DE ultrasonic unit used in the present study, with a maximum power of 250 W, was manufactured by Kunshan Ultrasonic Instruments Co. Ltd. (Kunshan, China). The unit was a cube-shaped container (23.5 × 13.3 × 10.2 cm), containing a 40 kHz ultrasonic transducer placed at the bottom. A circulating water-cooling system condensed the distillate continuously. A Ret-101 thermostatically-controlled water bath with a temperature controller (Neslab Instruments Inc., Newington, NH, USA) was connected to the ultrasonic unit to maintain the boiling state of the extracting solvent. The energy from the assembled UARSE device could thus be constantly transmitted into the reaction vessel.

### 3.3. Extraction Procedures

#### 3.3.1. Ultrasonic Assisted-Reflux Synergistic Extraction (UARSE)

A previous study has shown that methanol is a suitable solvent for extracting anti-cancer alkaloids from *C. acuminata* [29]. 3.0 g of the powdered dried fruit material with methanol (at liquid–solid ratios of 20, 25, 30, 35, and 40 mL/g) were placed in a 250-mL round-bottom flask and extracted by the UARSE apparatus (ultrasonic power settings of 150, 175, 200, 225, and 250 W; ultrasonic time of 15, 20, 25, 30, and 35 min), with the temperature of the water bath set at 75 °C. After UARSE, the extracts were cooled to room temperature then centrifuged for 10 min at 12,000 rpm (Heal Force Development Ltd., Hong Kong). The supernatants were then filtered through a 0.45 μm nylon membrane for subsequent HPLC analysis. Each procedure was repeated three times under the same conditions.

#### 3.3.2. Heating Reflux Extraction (HRE)

Based on the results of preliminary experiments, the extraction conditions were established as follows: 3.0 g of powdered dried materials were added to a round-bottom flask with 96 mL methanol, the flask was placed in a water-bath set at 75 °C, connected to a condenser, then extracted for 120 min.

#### 3.3.3. Ultrasonic-Assisted Extraction (UAE)

Based on the results of preliminary experiments, the extraction conditions were established as follows: 3.0 g of powdered dried sample were mixed with 96 mL methanol, put into a conical flask which was placed into the ultrasonic extraction device then extracted by sonication for 60 min at 45 °C at a power setting of 250 W.

### 3.4. Experimental Design of UARSE

RSM comprises a combination of mathematical and statistical approaches for optimizing experimental processes. After determining the preliminary range of extraction variables through single-factor tests, a three-level (−1, 0, +1), three-factor Box–Behnken design (BBD) combined with RSM was used to evaluate the main and interaction effects of the factors in the experimental region: 20–40 mL/g for the liquid–solid ratio (X_1_), 10–30 min for the ultrasonic time (X_2_) and 150–250 W for the ultrasonic power (X_3_) to allow the extraction yields for CPT (Y_1_) and BA (Y_2_) to be obtained. Table 1 presents the design matrix, which required a total of 17 experimental runs carried out in random order. Each run was performed in triplicate and the extraction yields were given as average values. The experimental data was fitted using the following second-order polynomial model:

(3) $$Y=\beta_{0}+\sum_{j=1}^{k}\beta_{j}X_{j}+\sum_{j=1}^{k}\beta_{jj}{X_{j}}^{2}+\sum \sum_{i<1}\beta_{ij}X_{i}X_{j}\;\left(k=3\right)$$

where *Y* represents the response variable, the extraction yield of each compound; *β*_0_, *β_j_*, *β_jj_*, and *β_ij_* are the regression coefficients of the variables for intercept, linearity, squared, and interaction terms, respectively; *X_i_* and *X_j_* are the independent variables influencing the response variable *Y*; and *k* represents the number of variables. The response surface and contour plots were constructed according to the fitted polynomial model. The experiment data was analyzed using response surface analysis software (Design-Expert 7.0.0 Trial, Stat-Ease Inc., Minneapolis, MN, USA). The analyses of variance (ANOVA) were performed to compare and determine the optimal conditions for UARSE.

### 3.5. HPLC Analysis

**Figure 8:**
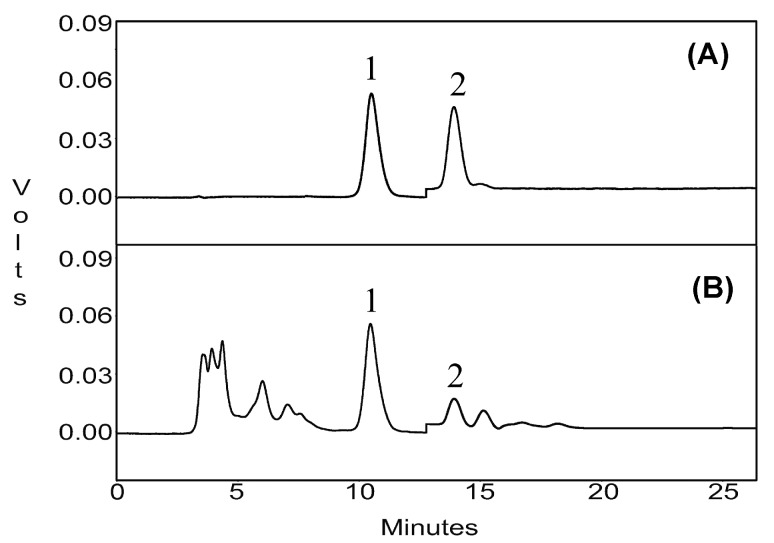
HPLC chromatograms for CPT and BA standards (**A**) and extract from *C. acuminata* fruits (**B**). Peak 1 for CPT and Peak 2 for BA.

The target compounds were quantified using an HPLC system consisting of a PU-980 pump, and an UV-975 detector (Jasco International Co. Ltd., Tokyo, Japan). Chromatographic separation was achieved on a Kromasil-C_18_ reversed-phase column (4.6 mm × 250 mm, 5 μm, KYA Technologies Corporation, Tokyo, Japan). The conditions for HPLC analysis were as follows: the mobile phase consisted of methanol: water (90:10, *v*/*v*), which was filtered through a 0.45 μm membrane filter, then deaerated ultrasonically before use; the flow rate was maintained at 0.8 mL/min; the injection volume was 20 μL; and the column temperature was 25 °C. The UV detection wavelength was set at 254 nm (0–13 min) then at 215 nm (13–25 min). The HPLC chromatograms of the standards and samples produced under these conditions are shown in Figure 8.

### 3.6. SEM Observation

The effect of the different extraction methods on the microstructure of the plant material was observed using scanning electron microscopy (SEM). The dried *Camptotheca acuminata* Decne. fruit samples and the samples obtained after UAE, HRE, and UARSE treatments were scanned using an electron microscope (Quanta-200 SEM, FEI Co., Hillsboro, OR, USA). The samples were fixed on aluminum stubs using adhesive tape then sputtered with gold using a sputter coater. All the samples examined were scanned under high vacuum conditions at an accelerating voltage of 12.5 kV (1000× magnification).

## 4. Conclusions

UARSE can be considered a novel and efficient method for extracting CPT and BA from *Camptotheca acuminata* Decne. fruits. On the basis of single-factor and BBD experiments, we selected the following optimized parameters: 32 mL/g for the liquid–solid ratio, 225 W for the ultrasonic power, and 24 min for the extraction time. The results indicated that UARSE had an obvious advantage in extraction yield over HRE and UAE (*p* < 0.01): the CPT and BA extraction yields obtained by UARSE were 2.386 ± 0.112 mg/g and 17.192 ± 0.808 mg/g, respectively, which were 1.43-fold and 1.33-fold higher compared with using HRE and UAE, respectively. In addition, the extraction time using UARSE was only 24 min, 80% and 60% less than that for HRE and UAE, respectively. This novel method has provided higher extraction yields than both HRE and UAE, suggesting that UARSE is an effective method for extracting CPT and BA from *Camptotheca acuminata* Decne. fruits. The UARSE method is also a promising method for extracting other useful natural products.

## Figures and Tables

**Table 1:** Experimental conditions used in the Box-Behnken design analysis and the corresponding measured responses.

Runs	Factors			Extraction Yield (mg/g)	
	X_1_ (mL/g) ^a^	X_2_ (min) ^b^	X_3_ (w) ^c^	CPT	BA
1	0(30)	0(20)	0(200)	2.333	16.448
2	0(30)	0(20)	0(200)	2.329	16.442
3	0(30)	−1(10)	−1(150)	1.591	11.607
4	−1(20)	0(20)	1(250)	1.693	13.366
5	1(40)	−1(10)	0(200)	1.498	14.434
6	0(30)	1(30)	−1(150)	1.552	13.374
7	−1(20)	−1(10)	0(200)	1.379	11.634
8	1(40)	0(20)	1(250)	1.983	15.968
9	1(40)	0(20)	−1(150)	1.525	11.939
10	0(30)	0(20)	0(200)	2.297	16.572
11	−1(20)	0(20)	−1(150)	1.344	10.822
12	0(30)	0(20)	0(200)	2.245	16.107
13	1(40)	1(30)	0(200)	1.611	15.828
14	0(30)	−1(10)	1(250)	1.885	14.861
15	0(30)	0(20)	0(200)	2.369	16.721
16	−1(20)	1(30)	0(200)	1.466	13.275
17	0(30)	1(30)	1(250)	2.385	16.883

^a^ X_1_ indicates the liquid–solid ratio (mL/g), ^b^ X_1_ the ultrasonic time (min), and ^c^ X_3_ the ultrasonic power (W).

**Table 2:** ANOVA of the response surface quadratic model for the yields of CPT and BA during the UARSE process.

Source ^a^	DF	CPT				BA			
		Sum of Square	Mean Square	*F* Value	*p*-Value ^b^	Sum of Squares	Mean Square	*F* Value	*p*-Value ^b^
***Model***	9	2.45	0.27	53.94	<0.0001	69.21	7.69	87.53	<0.0001
X_1_	1	6.80 × 10^−2^	6.80 × 10^−2^	13.38	0.0081	10.29	10.29	117.09	<0.0001
X_2_	1	5.50 × 10^−2^	5.50 × 10^−2^	10.82	0.0133	5.82	5.82	66.25	<0.0001
X_3_	1	0.47	0.47	92.63	<0.0001	22.23	22.23	253.02	<0.0001
X_1_X_2_	1	1.69 × 10^−4^	1.69 × 10^−4^	0.033	0.8600	1.50 × 10^−2^	1.50 × 10^−2^	0.17	0.6894
X_1_X_3_	1	2.97 × 10^−3^	2.97 × 10^−3^	0.59	0.4681	0.55	0.55	6.27	0.0407
X_2_X_3_	1	7.30 × 10^−2^	7.30 × 10^−2^	14.39	0.0068	1.60 × 10^−2^	1.60 × 10^−2^	0.19	0.6800
X_1_^2^	1	1.15	1.15	226.92	<0.0001	15.38	15.38	175.07	<0.0001
X_2_^2^	1	0.39	0.39	77.38	<0.0001	2.39	2.39	27.23	0.0012
X_3_^2^	1	0.10	0.10	20.51	0.0027	9.76	9.76	111.14	<0.0001
***Residual***	7	3.50 × 10^−2^	5.05 × 10^−3^			0.62	8.80 × 10^−2^		
Lack of Fit	3	2.70 × 10^−2^	8.89 × 10^−3^	4.11	0.1030	0.41	0.14	2.65	0.1847
*R^2^*		0.9858				0.9912			

^a^ X_1_ is the liquid–solid ratio (mL/g), X_2_ the extraction time (min), and X_3_ the microwave power (W). ^b^ *p* < 0.0001 is considered as significant.
